# A randomised controlled trial to assess the effectiveness of a single session of nurse administered massage for short term relief of chronic non-malignant pain

**DOI:** 10.1186/1472-6955-7-10

**Published:** 2008-07-04

**Authors:** Kate Seers, Nicola Crichton, June Martin, Katrina Coulson, Dawn Carroll

**Affiliations:** 1 RCN Research Institute, School of Health & Social Studies, University of Warwick, Coventry, CV4 7AL, UK; 2 Institute of Primary Care and Public Health, Faculty of Health and Social Care, London South Bank University, 103 Borough Road, London, SE1 0AA, UK; 3 Lymphoedema Clinic, Sir Michael Sobell House, Churchill Hospital, Old Road, Headington, Oxford, OX3 7LJ, UK; 4 Pain Management Department, Derby Hospitals NHS Foundation Trust, Derbyshire Royal Infirmary, London Road, Derby, DE1 2QY, UK; 5 Senior Health Outcomes Manager, Sanofi-aventis, Guildford, Surrey, UK

## Abstract

**Background:**

Massage is increasingly used to manage chronic pain but its benefit has not been clearly established. The aim of the study is to determine the effectiveness of a single session of nurse-administered massage for the short term relief of chronic non-malignant pain and anxiety.

**Methods:**

A randomised controlled trial design was used, in which the patients were assigned to a massage or control group. The massage group received a 15 minute manual massage and the control group a 15 minute visit to talk about their pain. Adult patients attending a pain relief unit with a diagnosis of chronic pain whose pain was described as moderate or severe were eligible for the study. An observer blind to the patients' treatment group carried out assessments immediately before (baseline), after treatment and 1, 2, 3 and 4 hours later. Pain was assessed using 100 mm visual analogue scale and the McGill Pain Questionnaire. Pain Relief was assessed using a five point verbal rating scale. Anxiety was assessed with the Spielberger short form State-Trait Anxiety Inventory.

**Results:**

101 patients were randomised and evaluated, 50 in the massage and 51 in the control group. There were no statistically significant differences between the groups at baseline interview. Patients in the massage but not the control group had significantly less pain compared to baseline immediately after and one hour post treatment. 95% confidence interval for the difference in mean pain reduction at one hour post treatment between the massage and control groups is 5.47 mm to 24.70 mm. Patients in the massage but not the control group had a statistically significant reduction in anxiety compared to baseline immediately after and at 1 hour post treatment.

**Conclusion:**

Massage is effective in the short term for chronic pain of moderate to severe intensity.

**Trial Registration:**

[ISRCTN98406653]

## Background

Complementary therapies, such as massage, are increasingly used by health professionals to manage a wide range of symptoms, including pain [1]. A literature search revealed a paucity of well-designed randomised controlled trials to determine the effectiveness of massage for chronic pain. This study aims to determine whether a single session of massage reduces short term pain and anxiety in patients with chronic pain.

The use of complementary therapies is steadily growing, both in Europe and in the US [2-4]. Health care practitioners are increasingly incorporating such therapies within their care [5]. This increased used is despite lack of evidence of effectiveness [6]. Health professionals' desire to deliver best practice is coupled with government initiatives to ensure an evidence based approach to care. For example, in the UK the National Institute for Clinical Excellence and NHS Quality Improvement Scotland have been set up; in the US the Agency for Health Care Research & Quality, and in Australia the National Institute for Clinical Studies all to try and ensure the health care delivered is based on what is known to be effective [7-10].

The holistic nature of nursing means massage is an attractive intervention for nurses to be able to offer patients. In addition to any direct therapeutic benefit, it allows nurses time with patients. It may be particularly relevant in settings where patients need immediate relief. Many nurses have undertaken courses in massage, however there is little robust evidence of its effectiveness. In today's evidence driven health care environment, if nurses are to justify the resources involved in training and delivery, they need evidence that massage is an effective treatment.

A literature search of Medline, EmBase, Cinahl and the Cochrane Controlled Trials Register and Cochrane Database of Systematic Reviews was undertaken, using free text and MeSH terms. Studies were sought which were randomised controlled trials, of manual massage therapies with or without oils, and which compared massage to at least one other treatment group (including usual care) in patients with chronic non malignant pain. Studies were excluded if they used non-manual massage or where massage was administered as part of a combination of therapies or was used for acute pain. Twenty eight studies were identified, of which eight met the criteria for inclusion.

After the RCT described in this paper was completed, a systematic review of massage for low back pain was published [11]. Outcomes considered in this review were pain, return to work, subjective change in symptoms and functional status. This superseded the literature review carried out prior to the conduct of the RCT reported in this paper. The systematic review concluded that massage might be beneficial for subacute and chronic non-specific low back pain, but that more studies were needed to confirm these conclusions [11]. Two of the RCTs included in review [11] found patients had less anxiety after massage [12,13]. Studies of chronic pain other than low back pain have found less anxiety and less pain [14-16].

In summary, despite the widespread and increasing use of massage for chronic pain, evidence of effectiveness is lacking, studies tend to be small and properly controlled studies are needed. An RCT to assess the effectiveness of massage for chronic pain was thus designed to add to the knowledge base in this area.

## Methods

### Aim

The aim of the study is to determine the effectiveness of a single session of nurse-administered massage for the short term relief of chronic non-malignant pain and anxiety.

### Design

A randomised controlled trial design was utilised, in which the patients were assigned to two groups by simple block randomisation, using blocks of 10. Randomisation was carried out by use of a computerised random number generator and provided from a central office unconnected with the study. After obtaining informed consent from the patient, the allocation was obtained by telephone from the central office, thus allocation was concealed.

### Sample and Setting

Patients aged 18 and over and attending a regional pain relief unit in England as in or out patients, who had experienced pain for three months or longer, whose pain was described as moderate or severe on the four point verbal rating pain intensity scale were eligible for the study. Patients were excluded if they did not speak English, did not consent or if they had taken any analgesics in the two hours prior to treatment. Outpatients were being followed up for the management of their chronic pain, and inpatients were admitted to the pain unit where patients stayed for one or more days in order to investigated ways of controlling their pain. The participants were a consecutive series of eligible patients and data collection took place between 1998 and 2000.

### Interventions

The experimental group received a 15 minute manual massage of their back, neck and shoulders using sweet almond oil. The control group received a 15 minute visit to talk about pain clinic treatment. The treatment for both the experimental and control groups was carried out by two registered nurses qualified in massage (ITEC diploma). Each therapist (JM, KC) performed a sequence of the same massage techniques with the patients in the massage treatment group. This involved effleurage, petrissage and kneading techniques. For patients in the control group the therapist visited the patient for 15 minutes and encouraged them to talk about their pain and pain treatment. This is representative of usual care in this pain clinic, where health care staff talk to patients about their pain and its management, and no massage was performed for the control group. Patients in both groups were asked to try and complete at least one full hour after the intervention before requesting analgesics, but could request analgesics at any time after the intervention. If they received an analgesic, they were then excluded from any further follow-up assessments.

### Outcomes

An independent nurse observer (DC), blind to the patients' treatment group, carried out assessments of the patient. These were carried out immediately before treatment (baseline), immediately after treatment (post treatment) and at 1, 2, 3 and 4 hours after treatment. The observer also recorded whether or not the patient had broken the blinding by inadvertently revealing their group allocation (for example, by referring to "my massage"). Once blinding was broken, it was considered unblinded for all subsequent assessments for that patient.

Pain intensity was assessed using a 0–100 mm visual analogue scale (VAS), the four point verbal rating pain intensity scale with descriptors of none = 0, mild = 1, moderate = 2 and severe = 3, both have been shown to be reliable, valid and appropriate for clinical use [17], and The McGill Pain Questionnaire Pain Rating Index [18]. Pain Relief was assessed using five point verbal rating scale with descriptors of none = 0, slight = 1, moderate = 2, good = 3 and complete relief = 4. Anxiety was assessed with the six item Spielberger short form State-Trait Anxiety Inventory, [19]. At the end of the study period patients made an overall rating of the study treatment using a five point scale (poor = 0, fair = 1, good = 2, very good = 3 or excellent = 4). They also rated whether they had achieved 50% pain relief [20].

### Sample Size

The sample size was calculated for comparing mean difference in pain score and for the study to have a power of 90% [21]. The results of a study of pain relief through relaxation in elderly hip fracture patients suggests that when using a pain scale rating pain from 1 to 10 that the standard deviation for patients with chronic pain on this scale is 2.5 [22]. On this scale a mean difference of 1.5 in pain scores between the treatment groups could be considered clinically worthwhile. Since in the current study a 100 mm VAS pain scale will be used a standard deviation of 25 mm and a mean difference of 15 mm are consistent with the earlier study [22]. With type I error set at 0.05, then the study would require 60 patients per group. If a difference as large as 20 mm between the pain scores was attained this could be demonstrated with 34 patients per group. In the current study it was decided, based on the difference found in the previous study of elderly hip fracture patients, recruit 60 to each group: a total of 120 patients.

### Ethical considerations

The study received ethical approval from the Local Research Ethics Committee (Study Code NAPREC 97.053). All participants recruited to the study had received a verbal explanation of the study and were provided with a written information sheet. All potential participants had at least 24 hours between receiving information and deciding whether or not they wished to take part in the study. All participants gave written consent.

### Data analysis

In the analysis baseline pain and anxiety scores are compared for the two treatment groups using t-tests. To investigate the treatment effect, for each patient their pain scores after treatment are compared to their baseline pain score (pre-treatment). The mean difference in change in pain score for the two treatment groups is compared using a 2 sample t-test. Similarly changes in anxiety between baseline and post treatment are compared for the two groups sample t-test. Box plots are used to show graphically the differences between groups. The box includes the 25^th^, 50^th ^and 75^th ^percentiles; the whiskers indicate the range of the data, with asterisks indicating outliers. Statistical significance was set at the 5% level. The data were analysed using SPSS version 12 and MINITAB version 13.

## Results

In total 103 patients participated in the study of whom 52 were randomly allocated to the massage group and 51 to the control group. Two patients allocated to the massage group withdrew at baseline, one because they received morphine just prior to starting the study and the other because they were unable to understand the assessment scales, thus the analysis reports results for the 101 patients who completed the baseline assessments. The patients were aged between 21 and 81 years, with mean age 53.4 years (SD 13.3 years) and 58.4% were female. The mean time that the patients had experienced chronic pain was 10.4 years (SD 8.94 years). 87% of the patients had a diagnosis which included back pain and 58% had more than one pain site. At baseline, 60.4% had moderate and 39.6% had severe pain as recorded on the verbal rating scale.

**Figure 1:**
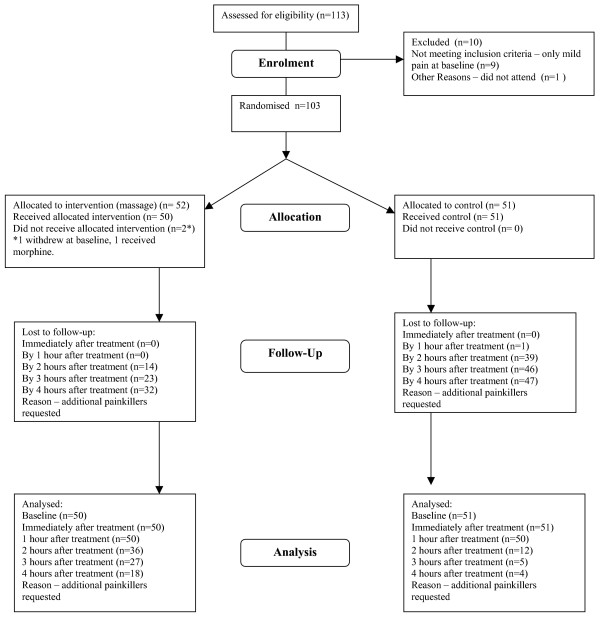
Participant Flow at each stage of study.

Figure 1 shows the number of patients still taking part in the study at each assessment point. After the first hour the drop out from both groups, but particularly from the control group was rapid. By the 2 hour post-treatment assessment only 23% of patients in the control group remained in the study and by the 3 hour assessment only 10% of the control group were available for assessment. In comparison for the massage group 72% of patients remained in the study at 2 hours post-treatment, 54% at 3 hours and 36% at 4 hours post treatment. Because of the very high withdrawal rate at 2, 3 and 4 hours after treatment, the results from these time periods are impossible to interpret and are therefore not presented.

The assessment of the patients at all time points was by an observer who was blind to the treatment that the patient received (DC). Occasionally the patient would break the blinding by comments that they made to the observer. For 89 of the 100 patients in which a record of blinding was made, the observer remained blind throughout the assessment, so blinding was maintained in 89% of patients.

The demographic characteristics and baseline scores for the massage and control groups are compared in Table 1. Comparisons between massage and control groups were tested using chi-squared or two sample t-tests as appropriate. There are no significant differences in the age, gender mix or duration of pain for the two treatment groups.

**Table 1:** Comparison of the characteristics of the massage and control groups at baseline (pre-treatment)

**Characteristic**	**Massage group**	**Control Group**	**p-value**
Number in group	50	51	
Gender (male:female)	22:28	20:31	0.626 NS
Age in years Mean (SD)	51.5 (13.4)	55.2 (12.9)	0.171 NS
Duration of pain in years. Mean (SD)	9.86 (7.47)	11.0 (10.2)	0.540 NS
Therapist (A:B)	27:23	27:24	0.915 NS
Pain VAS. Range 0–100. Mean (SD)	57.7 (18.0)	62.3 (16.6)	0.200 NS
McGill Pain Q'aire Pain Rating Index Range 0–78 Mean (SD)	27.3 (11.5)	25.1 (14.4)	0.390 NS
Spielberger *SF *STAI Range 6–24 Mean (SD)	13.3 (4.16)	12.0 (4.15)	0.139 NS

**Figure 2:**
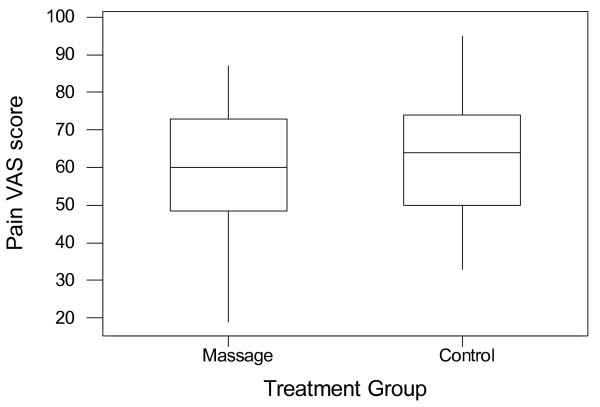
Baseline (pre-treatment) pain VAS scores for the massage and control groups.

The pain verbal rating scale scores are "moderate" or "severe" for all patients, with 40% of the massage group and 39% of the control group recording severe pain at baseline. The similarity of the distribution of pain scores between the two groups is shown in the boxplot in Figure 2.

**Figure 3:**
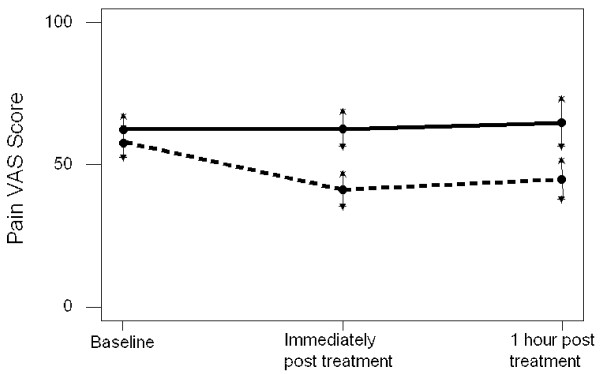
Mean and 95% confidence interval for the mean pain VAS scores for the massage group (- - - - -) and the control group (―) at three time points.

Comparisons post-treatment of pain VAS scores are shown in Figure 3 and Table 2. Figure 3 illustrates that the 95% confidence intervals for mean pain VAS for the massage and control groups overlap at baseline, the means are not significantly different. However, the confidence intervals are clearly distinct both immediately post treatment and at 1 hour post treatment, consistent with the massage group having significantly lower mean pain VAS. For the control group mean pain VAS for those who continue beyond 1 hour (the minority) is 36.4, but mean pain VAS for those in the control group who withdraw at 1 hour (the majority) is 74.7, a significantly higher mean (p < 0.001).

**Table 2:** Comparison of massage group and control group for both pain VAS scores and changes in pain VAS score from baseline, at post treatment assessments*.

**Time point**	**Massage group**			**Control group**			**p-value (2 sample t-test)**
		
	Mean	SD	N#	Mean	SD	N#	
Baseline score	57.7	18.0	49	62.3	16.6	47	0.200 NS

Immediately post treatment score	41.1	20.2	49	62.8	20.9	47	
Change from baseline	16.7	21.2	48	-0.04	16.0	45	0.000

1 hour post treatment score	44.8	23.5	47	64.9	26.7	43	
Change from baseline	12.4	21.3	47	-2.66	24.0	41	0.002

* Note that at each time point adding the change from baseline scores to the post treatment scores will not necessarily equal the baseline scores, because some patients have dropped out after the baseline

SD = Standard Deviation

NS = Not significant

# the table has some missing data on VAS scores at baseline, but data available on other outcomes. One participant in the massage group and 3 in control group did not complete VAS pain scores, and thus were not included in the later comparisons to baseline.

Table 2 compares the baseline score to that immediately post treatment, the massage group experience a statistically significant (p < 0.001 using paired t-test) mean reduction in pain of 16.7 mm (SD 21.2). For the control group the mean change in pain score is -0.04 mm (SD 16.0) which is not significant (p = 0.985 using paired t-test). For those in the massage group there is a significantly greater reduction in pain score both immediately post treatment and at 1 hour post treatment than in the control group. The 95% confidence interval for the difference in mean pain reduction at one hour post treatment between the massage and control groups is 5.47 mm to 24.70 mm, that is the massage group can expect on average to benefit by between 5.47 mm and 24.70 mm greater reduction than the control group up to one hour post treatment.

**Figure 4:**
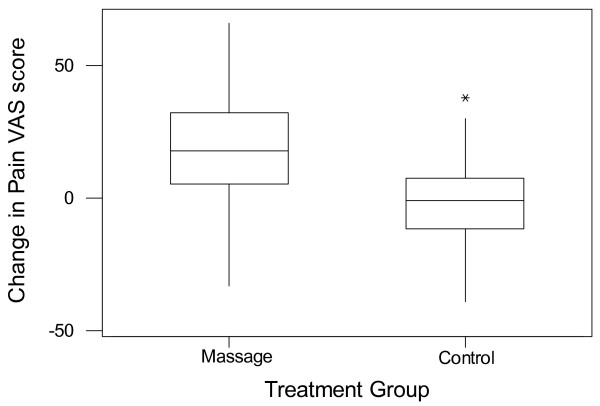
**Change in pain VAS score between baseline and immediately post treatment for both the massage group and the control group.** The change is calculated as baseline – post-treatment, so positive values indicate pain has been reduced by treatment.

Figure 4 illustrates the reduction in pain VAS score for the massage group and the negligible change in pain VAS score for the majority of members of the control group. For both groups the median is central in the box of the boxplot, thus supporting the assumption that the data is normally distributed and that a t-test can be used to compare these changes.

The results from the four point verbal rating pain, the four point pain distress scale and the McGill Pain Questionnaire produce the same findings as the pain VAS scale. That is, statistically significant differences between massage and control groups immediately and one hour post treatment.

An example of these similarities is shown in comparisons of McGill Pain Questionnaire scores from baseline to immediately post treatment. The massage group experience a statistically significant (p < 0.001 using paired t-test) mean reduction in McGill pain score of 11.5 (SD 9.93). For the control group the mean change in McGill pain score is 1.22 (SD 7.16) which is not statistically significant (p = 0.237 using paired t-test).

Table 3 showed, using a chi-squared test, a significant association between treatment group and whether the patient gets 50% pain relief, with only one person in the control group but 18/31 and 18/32 patients in the massage group reporting 50% pain relief immediately post and 1 hour post treatment.

**Table 3:** Comparison of massage group and control group for 50% pain relief at post treatment assessment.

**Time point**	**Massage group 50% pain relief**		**Control group 50% pain relief**		**p-value**
	
	Yes	No	Yes	No	
Immediately post treatment Change from baseline	18	31	0	51	0.000
1 hour post treatment change from baseline	18	32	1	49	0.000

Table 4 illustrates results from the Spielberger short-form anxiety scale. It shows that comparing the baseline score to that immediately post treatment, the massage group experience a statistically significant (p < 0.001 using paired t-test) mean reduction in STAI score of 3.57 (SD 3.02). For the control group the mean change in STAI score is 0.00 (SD 3.39) which is not statistically significant (p = 1.00 using paired t-test). For those in the massage group there is a statistically significantly greater reduction in anxiety score both immediately post treatment and at 1 hour post treatment than in the control group. The 95% confidence interval for the difference in mean anxiety score reduction at one hour post treatment between the massage and control groups is 1.67 to 4.36 that is the massage group can expect on average to benefit by between 1.67 and 4.36 greater reduction in anxiety score than the control group up to one hour post treatment.

**Table 4:** Comparison of massage group and control group for both Spielberger STAI scores and changes in STAI score from baseline, at post treatment assessments.

**Time point**	**Massage group**			**Control group**			**p-value**
		
	Mean	sd	n	Mean	Sd	N	
Baseline score	13.3	4.16	50	12.0	4.15	47	0.139 NS

Immediately post treatment score	9.72	3.43	47	12.2	5.15	47	
Change from baseline	3.57	3.02	47	0.00	3.39	44	0.000

1 hour post treatment score	10.5	3.90	50	12.8	5.06	47	
Change from baseline	2.74	3.10	50	-0.28	3.43	43	0.000

At the final assessment for the patient, either at 4 hours post treatment or at the point that they requested an analgesic and thus withdrew from the study the patient was asked to assess whether the treatment they had received was poor, fair, good, very good or excellent. In the massage group, 34 rated the treatment as good, very good or excellent, 12 as fair and 4 as poor. In the control group, 4 rated it as good, very good or excellent, 14 as fair and 33 as poor. A chi-squared test for the patients' assessment of the treatment demonstrates that those receiving the massage rate this treatment statistically significantly better than the control group (X^2 ^= 46.6, df = 2, p < 0.001).

At the final assessment the patient was asked whether they would like to have the treatment again. A significantly higher proportion of those in the massage group (X^2 ^= 24.4, df = 1, p < 0.001) said they would like the treatment again. Of the 49 patients in the massage group who were asked this question all 49 (100%) said yes they would like the treatment again. Of the 42 patients in the control group asked this question, 25 (60%) said yes they would like the treatment again.

## Discussion

This study showed that, compared to baseline, massage reduced pain and anxiety for up to one hour. There were no such differences for the control group. This suggests that massage could be a useful short term intervention, to reduce both pain and anxiety. Although an hour's pain relief after a massage may seem a short duration, this could be very worthwhile if, for example, the patient is anxious or fearful about a procedure they are about to undergo, or they are waiting for an analgesic to take effect, or to give them some short term respite from pain during the day and thus a sense of a degree of control over the pain.

The results showed that these patients with moderate to severe chronic pain are used to taking analgesics, with 77% of patients in the control group dropping out to take an analgesic after one hour post intervention, and 92% control patients had dropped out by 4 hours post intervention.

One of the problems of demonstrating any statistically significant effect of massage over time was the large drop out rate. Once the patient had taken an analgesic, the effects due to the massage could no longer be reliably elicited and they had to be excluded. The differential drop out rate between the groups is interesting. At 2 hours post treatment 72% of patients having massage compared to only 23% of those in the control group remained in the study. At 3 hours post treatment 53% of massage patients compared to only 10% of control patients remained. At the final assessment after 4 hours, over a third (36%) of massage patients remained compared to only 8% of those in the control group. This gives at least an indication that because patients who dropped out did so because they requested an analgesic, those in the massage group who stayed in the study did so because they had less pain.

The study had aimed to recruit 120 patients, 60 in each group. In the event, 101 were included in the study. This was based on a previous study of relaxation in hip fracture patients which had a standard deviation of 25 mm and a mean difference of 15 mm [18]. If we take the actual VAS pain scores comparing baseline to immediately post treatment, there is a mean difference of 16 mm and a standard deviation of 21 mm. For a mean difference and standard deviation of this size a sample size of 45 per group, gives power of 95%. So, looking at this outcome, the study is adequately powered.

The extent of a statistically significant reduction of pain and whether it is clinically significant is important to consider. Compared to baseline, those in the massage group had a mean reduction in pain of 16.7 mm on a 0–100 scale immediately after treatment. If 50% pain relief is taken as clinically important, 36% of the massage group reported at least this level of relief immediately post treatment, compared to 0% of the control group. This provides some evidence that the massage can have quite a large effect in just over a third of patients. Although a benefit of reduced pain for one hour may seem a limited effect, it does provide something of a break from pain.

It is then a clinical question whether the 15 minute investment of time in doing the massage is worth the benefits outlined in the paper. When patients either completed the study or withdrew, 68% of massage patients compared to only 8% of control patients said the intervention had been good, very good or excellent, so it would seem over two-thirds of patients did find the treatment beneficial. This could be one area where carers may be able to take on this role, although anecdotally, we are aware that some clinicians may be reluctant to make this suggestion to carers, fearing litigation if there are any adverse effects. Health professionals wishing to integrate massage into their practice need to consider professional training, and the requirements of the regulatory bodies of their own country. For example, in the UK, the Royal College of Nursing produced guidance on integrating complementary therapies into clinical care [23]. It would also be sensible to liaise with the appropriate committees within their own hospital or community setting regarding the level of training required to practice (for example, the formal structures addressing risk management, quality assurance and professional development).

This study adds to the international knowledge base about the effectiveness of massage for chronic pain. It has demonstrated that massage can produce short term reductions in pain and anxiety, and improvements in pain relief which are valued by patients. These are important potential benefits for people in whom the management of pain is often a challenge.

## Conclusion

Massage is effective in the short term for chronic pain of moderate to severe intensity, and has a small anxiety reducing effect. It could be a useful addition to techniques offered to patients as part of their care. Nurses are well placed to be trained in and to deliver massage. More research is needed to explore the benefits of repeated treatments of massage in patients with chronic pain.

## Competing interests

The authors declare that they have no competing interests.

## Authors' contributions

KS conceived the study and participated in its design and coordination and drafted the manuscript. NC participated in the design, conducted the statistical analysis and helped to draft the manuscript, JM and KC were involved in the data collection, delivering the intervention and helped draft the manuscript, DC participated in the design of the study and the data collection and helped draft the manuscript. All authors read and approved the final manuscript.

## Pre-publication history

The pre-publication history for this paper can be accessed here:


